# Age-Related Mortality Trends in Italy from 1901 to 2008

**DOI:** 10.1371/journal.pone.0114027

**Published:** 2014-12-08

**Authors:** Marina Vercelli, Roberto Lillini, Alberto Quaglia, Rosanna T. Micale, Sebastiano La Maestra, Silvio De Flora

**Affiliations:** 1 Department of Health Sciences, University of Genoa, Genoa, Italy; 2 Descriptive Epidemiology Unit, IRCCS Azienda Ospedaliera Universitaria San Martino - Istituto Nazionale per la Ricerca sul Cancro (IST), Genoa, Italy; 3 Vita-Salute San Raffaele University, Milan, Italy; University of California, United States of America

## Abstract

We stratified the Italian population according to age and gender in order to evaluate mortality trends over more than one century. Data covering the 1901–2008 period were used to study the yearly variations in mortality. Fluctuations in age-adjusted mortality curves were analyzed by Join Point Regression Models, identifying Join Points and Annual Percent Changes. A consistent decline in all-cause mortality occurred across the whole period, the most striking variations being observed in the 0–49 years population. In 1901, other and undefined diseases were the main causes of death, followed by infectious, digestive, and respiratory diseases in the 0–49 years population and by respiratory, cardiovascular, and cerebrovascular diseases in the ≥50 years population groups. In 2008 the main causes of death were accidents (males) and tumors (females) in the 0–49 age class, tumors in the 50–69 age class (both genders), and tumors (males) and cardiovascular diseases (females) in the elderly. The results highlight the interplay between age and gender in affecting mortality trends and reflect the dramatic progress in nutritional, lifestyle, socioeconomic, medical, and hygienic conditions.

## Introduction

The “epidemiological transition” theory was first described in 1971 [1] to illustrate the dynamic relationship between epidemiological trends and demographic patterns during the first 7 decades of the 20^th^ century. More recently, when displaying the dramatic crossover of mortality curves by mid 20th century in Italy, we introduced the concept of “epidemiological revolution”, which is chiefly characterized by the replacement of infectious and parasitic diseases with chronic degenerative diseases (CDDs) as the main causes of death in the population [2]. In fact, CDDs have become the leading causes of death in all world regions except Africa. However, current projections indicate that by 2020 the largest increases in CDD deaths will occur in that continent, where in 2030 they will exceed the combined deaths of communicable and nutritional diseases and maternal/perinatal deaths as the most common cause of death [3], [4]. Although the term CDD is not particularly attractive, we prefer it to the terms “chronic diseases”, since certain infectious diseases tend to become chronic as well, and “noncommunicable diseases”, since a fair proportion of CDDs, such as cancers [5], have an infectious origin.

The “epidemiological revolution” is related to fundamental advances in biomedicine, and especially to the discovery of pathogens responsible for communicable diseases, as well as to changes in medical care, environment, and lifestyle. In addition, the impressive aging of the population and increase in the proportion of elderly people contributed to the escalation of CDDs. However, by using age-adjusted data rather than crude data, a decrease in the mortality for CDDs became evident in Italy during the last decades of the 20^th^ century, relatively not only to cardiovascular and cerebrovascular diseases but even to cancers [2]. Thus, the epidemiology of the main causes of death in the population will evolve in any region in the world through three consecutive stages, including (*a*) a period lasting centuries or millennia during which infectious diseases are the plagues that decimate the population, (*b*) a period during which there is a dramatic crossing of the mortality curves for infectious diseases and CDDs, and (*c*) a further turning point after which even mortality for CDDs tends to decrease, with obvious benefits on all-cause mortality. In Italy and several other countries this 3-stage evolution in the epidemiologic scenario occurred within the 20^th^ century [2].

Recently, we extended the analysis of demographic and mortality data in Italy, year by year, from 1901 to 2008 [6]. This is one of the longest periods ever evaluated in terms of population growth, live birth rates, life expectancy at birth, infant mortality and all-cause mortality rates, and mortality for the main causes of death. In the present study, we stratified the all-cause mortality data and mortality for the 10 main causes of death in the Italian population from 1901 to 2008 according to age groups. The emerging results highlight sharp age-related differences in mortality curves in both genders and in the contribution of different death causes to the overall mortality.

## Methods

### Mortality Database

Mortality data in the Italian population from 1901 to 2008 were available by gender and quinquennial age classes from the Italian Institute of Statistics [7]–[9] (see S1 Appendix). In addition to all-cause mortality, the following seven main death causes were evaluated over the whole period: infectious and parasitic diseases (INF; categories A00 – B99 according to ICD-10), malignant tumors (TUM; C00 – D09), cardiovascular diseases (CV; I00 – I52; I70 – I99), cerebrovascular diseases (CBV; I60 – I69), respiratory diseases, also including influenza (RESP; J00 – J99), digestive system diseases (DIG; K00 – K93), and accidents (ACC; V01 – Y98). Two further main causes of death entered the database in 1951: endocrine, nutritional and metabolic diseases, also including AIDS, which was added in 1982 [10] (END; E00 - E90; B20-B24) and the nervous system diseases (NS; G00 - G99). The tenth evaluated cause included all remaining deaths, identified as other and undefined causes of death (OUC). The frequent revisions of ICD were already taken into account in WHO and ISTAT databases [11], [12]. Inclusion of AIDS in the END group of diseases, due to the late adoption of ICD-10 coding by ISTAT since 2003, was the only adjustment needed.

### Stratification by Gender and Age Class

All mortality data were stratified by gender in three broad age classes: young (0–49 years), middle aged (50–69), and elderly (≥70). For these three groups, the Standard Mortality Rates (SMR) were computed for every year of the whole period by gender, using the age-specific rates and referring to the World Population age distribution standard proposed by Segi [13], [14].

The three age groups were selected on the basis of clinical, biological, epidemiological and statistical reasons. The choice takes into account the recent situation in the populations of developed countries, where the definition of “elderly” has been raised to 70 years and over, compared to quite different conditions at the beginning of the twentieth century. The age group 50–69 was chosen in order to differentiate the group of older people, having major problems of physical decay, from adults approaching the old age, who are facing major preventive interventions for CDDs. The youngest age group (0–49) was chosen because of the low number of deaths in the second half of the examined period and, particularly, in the light of the contrast between communicable diseases and CDDs. In fact, from a statistical point of view, the deaths after 1950 were severely reduced in all the layers of this age class, making the rates for specific causes of death very small and with very broad confidence intervals. The standardization carried out within the three groups provides a good balance of the effects produced by the layers of 5 years. The chosen age groups have been used worldwide in a number of epidemiological studies regarding the epidemiological transition [15] and the leading causes of death in the population [16], such as cardiovascular diseases [17], [18] and cancer [19]–[22].

### Join Point Regression Model

Within each gender, age class, and cause of death, we analyzed the time-related fluctuations in death rates by means of Join Point Regression model (Join Point Linear Regression or JPLR) [23], which allowed us to detect the years in which there are variations in the slope of mortality curves, identified as Join Points (JPs), and the average Annual Percent Change (APC) between two consecutive JPs. Details on the JPLR are given in S2 Appendix and [24]. All analyses were performed by Join Point Regression 3.3.1 and SPSS 15.0 software. The statistical threshold for the JP model was fixed at *P*<0.05.

### Ethics Statement

Only publicly available data were analyzed. No patient records/information was available, but only a matrix of data by year and age of death.

## Results

### General Outline of Results

The complete JPLR results are attached as Excel files compressed in “JP regression results.rar” archive (S1 File). The compressed archive can be opened with WinRAR software or other similar compressing/decompressing software. The archive contains 11 Excel files that report all the regression results as age-standardized death rates for males and females, separately for the three age classes.

### All-Cause Mortality Trends from 1901 to 2008

**Figure 1:**
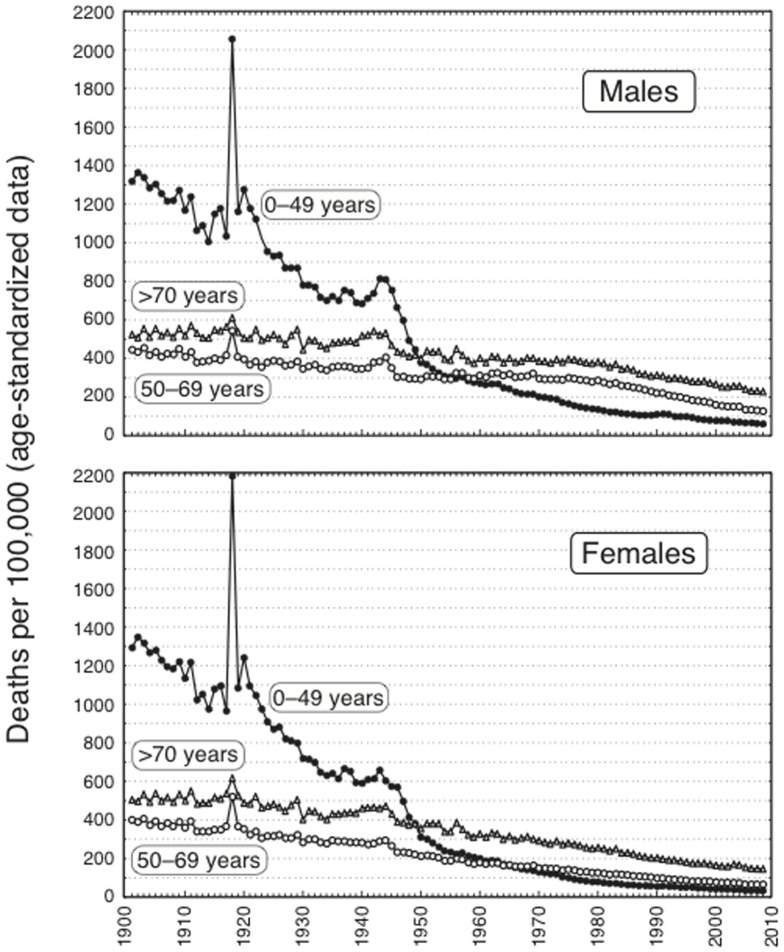
Age-standardized all-cause mortality in Italy from 1901 to 2008 as related to gender and age classes.

Fig. 1 shows the curves depicting all-cause mortality data covering more than one century (1901–2008) of Italian history. It should be noted that JPLR detects regular trends but does not take into account peaks, such as those due to the Spanish flu (1918) and the Second World War (1941–1944), which are well evident in Fig. 1. A consistent trend to a decline in mortality occurred across the whole period, with a pace varying depending on gender and age. In particular, as detailed in Table 1, the most striking variations were observed in the 0–49 years population, in which the JPLR analyses revealed similar trends in males and females, highlighting in both genders a strong decrease during the first half of the 20^th^ century, until 1946, followed by an even sharper decrease until 1950–1952 and by a steady decline until 2008. Given the large heterogeneity in the 0–49 age group throughout the century, S1 Figure shows the contribution of age sub-groups to the all-cause mortality and cause-specific mortality by using the proportional mortality with respect to the all ages general mortality.

**Table 1:** Age-adjusted mortality rates (per 100,000, world standard), for all causes and specific causes, and Annual Percent Changes (APC) between Join Points (JP) in Italian males (A) and females (B) aged 0–49 years during the period 1901–2008.

Causes of death	1901–2008 APC	Start (year)	APC1 (95% CI)	1^st^ JP (year)	APC2 (95% CI)	2^nd^ JP (year)	APC3 (95% CI)	3^rd^ JP (year)	APC4 (95% CI)	End (year)
**A. Males**										
ALL	−3.2*	1318.3 (1901)	−1.7* (−1.9, −1.4)	661.2 (1946)	−13.9* (−24.7, −1.5)	379.4 (1950)	−3.1* (−3.2, −2.9)			60.9 (2008)
RESP	−5.8*	278.2 (1901)	−1.4* (−2.0, −0.8)	177.2 (1939)	−7.9* (−8.2, −7.5)	1.9 (1993)	−4.1* (−6.5, −1.6)			1.2 (2008)
DIG	−5.0*	276.3 (1901)	−1.0* (−1.6, −0.3)	262.7 (1922)	−2.7* (−3.3, −2.1)	119.0 (1946)	−13.3* (−16.4, −10.0)	34.9 (1954)	−5.2* (−5.4, −5.1)	2.6 (2008)
INF	−6.6*	268.0 (1901)	−2.0* (−2.3, −1.8)	122.3 (1946)	−16.8* (−24.3, −8.5)	48.8 (1951)	−10.0* (−10.3, −9.7)	0.8 (1990)	+2.0* (+1.0, +3.0)	1.0 (2008)
CV	−1.4*	31.4 (1901)	+0.9* (+0.2, +1.5)	45.1 (1918)	−5.5* (−9.2, −1.5)	22.8 (1924)	−0.6* (−0.8, −0.5)	19.9 (1966)	−2.6* (−2.7, −2.4)	6.4 (2008)
CBV	−1.7*	10.2 (1901)	−0.7* (−1.0, −0.4)	7.5 (1929)	−2.5* (−2.9, −2.1)	3.8 (1955)	+0.2 (−0.2, +0.6)	3.9 (1980)	−4.3* (−4.6, −3.9)	1.3 (2008)
TUM	+0.5*	9.0 (1901)	+0.8* (+0.7, +1.0)	14.4 (1949)	+10.7 (−10.1, +36.2)	19.0 (1952)	+0.9* (+0.5, +1.4)	23.3 (1977)	−2.5* (−2.8, −2.3)	11.0 (2008)
ACC	−0.6*	40.8 (1901)	−0.1 (−0.3, +0.1)	37.0 (1941)	+34.6* (+5.0, +72.7)	115.0 (1944)	−22.3* (−39.4, −0.4)	47.6 (1947)	−1.2* (−1.3, −1.1)	20.0 (2008)
END	−0.2#	7.5 (1951)	−4.9* (−5.2, −4.6)	1.8 (1983)	+21.0* (+19.1, 23.0)	12.0 (1995)	−30.7* (−38.6, −21.8)	3.5 (1999)	−1.1 (−3.2, +1.1)	2.9 (2008)
NS	−2.8#*	8.2 (1951)	−1.0* (−1.8, −0.2)	6.9 (1963)	−4.3* (−4.7, −3.9)	3.1 (1983)	−1.5* (−1.7, −1.2)			1.9 (2008)
**B. Females**										
ALL	−3.9*	1292.9 (1901)	−2.1* (−2.3, −1.9)	570.1 (1946)	−12.1* (−17.9, −5.8)	281.1 (1952)	−3.8* (−3.9, −3.6)			32.8 (2008)
RESP	−6.2*	237.4 (1901)	−1.7* (−2.4, −1.0)	138.2 (1940)	−8.1* (−8.4, −7.8)					0.7 (2008)
DIG	−6.2*	272.7 (1901)	−2.1* (−2.4, −1.9)	111.1 (1945)	−13.7* (−16.5, −10.8)	27.4 (1954)	−7.7* (−8.0, −3.3)	2.3 (1988)	−4.6* (−5.4, −3.8)	0.9 (2008)
INF	−7.3*	293.0 (1901)	−2.5* (−2.7, −2.3)	109.0 (1946)	−18.3* (−22.3, −14.1)	24.7 (1953)	−10.0* (−10.2, −9.7)	0.5 (1992)	+1.9* (+0.8, +3.1)	0.5 (2008)
CV	−3.0*	40.5 (1901)	−1.2* (−1.4, −1.1)	22.0 (1949)	−4.8* (−5.0, −4.5)	3.3 (1990)	+3.1 (−2.1, +8.5)	3.7 (1996)	−5.7* (−7.0, −4.4)	2.0 (2008)
CBV	−1.9*	8.3 (1901)	−1.1* (−1.3, −0.9)	5.0 (1946)	−6.3* (−10.8, −1.7)	3.1 (1952)	−1.2* (−1.4, −1.0)	2.0 (1994)	−6.7* (−7.6, −5.7)	0.9 (2008)
TUM	−0.1*	16.2 (1901)	−0.2* (−0.3, 0)	15.8 (1945)	+2.2* (+1.6, +2.9)	21.2 (1960)	−1.0* (−1.2, −0.8)	16.8 (1987)	−1.9* (−2.3, −1.6)	11.3 (2008)
ACC	−1.1*	17.7 (1901)	−0.5* (−0.8, −0.3)	14.6 (1946)	−8.4 (−21.3, +6.7)	10.1 (1950)	+0.3 (−0.3, +0.9)	11.0 (1976)	−2.3* (−2.7, −1.9)	4.6 (2008)
END	−2.0#*	6.2 (1951)	−5.2* (−5.6, −4.9)	1.3 (1984)	+13.5* (+11.1, +16.0)	4.4 (1995)	−23.3* (−33.2, −11.9)	1.7 (1999)	−1.6 (−4.1, +0.9)	1.6 (2008)
NS	−3.2#*	6.4 (1951)	−1.1 (−2.1, 0)	5.5 (1963)	−4.9* (−5.4, −4.4)	1.8 (1984)	−1.0* (−1.8, −0.1)	1.7 (1999)	−4.2* (−5.8, −2.6)	1.9 (2008)

*P<0.05; #Period 1951–2008.

The curves started from much lower levels in the two older population groups. Mortality curves in the middle aged and elderly population groups were roughly parallel and crossed the mortality curves for 0–49 years by mid 20th century (Fig. 1). Tables 1, 2, and 3 report details on age-adjusted mortality rates, for all causes and specific causes, and the APCs between JPs in the populations aged 0–49, 50–69, and ≥70 years, respectively. On the whole, in 2008 the all-cause mortality ratios in males to females were 1.9 in subjects aged 0–49 and 50–69 *vs.* 1.5 in subjects aged ≥70. Thus, starting from similar levels in the two genders at the beginning of the 20th century, in 2008 mortality was almost the double in males compared to females of less than 69 years, whereas the inter-gender differences were less pronounced in older people.

**Table 2:** Age-adjusted mortality rates (per 100,000, world standard), for all causes and specific causes, and Annual Percent Changes (APC) between Join Points (JP) in Italian males (A) and females (B) aged 50–69 years during the period 1901–2008.

Causes of death	1901–2008 APC	Start (year)	APC1 (95% CI)	1^st^ JP (year)	APC2 (95% CI)	2^nd^ JP (year)	APC3 (95% CI)	3^rd^ JP (year)	APC4 (95% CI)	End (year)
**A. Males**										
ALL	−0.9*	446.2 (1901)	−0.6* (−0.6, −0.5)	259.6 (1984)	−3.1* (−3.4, −2.8)					126.6 (2008)
RESP	−2.9*	109.8 (1901)	−1.5* (−1.9, −1.1)	62.9 (1939)	−7.8* (−9.7, −5.8)	21.5 (1952)	+1.9* (+0.5, +3.3)	37.3 (1969)	−4.9* (−5.3, −4.6)	4.0 (2008)
DIG	−1.0*	40.7 (1901)	−2.7* (−4.0, −1.4)	27.8 (1913)	−0.3* (−0.6, −0.1)	25.1 (1955)	+1.0* (+0.4, +1.5)	29.5 (1978)	−5.1* (−5.4, −4.8)	7.0 (2008)
INF	−3.7*	36.4 (1901)	−0.3* (−0.5, −0.1)	33.9 (1944)	−4.2* (−5.0, −3.5)	12.7 (1963)	−9.0* (−9.4, −8.6)	0.9 (1991)	+5.2* (+4.3, +6.1)	1.9 (2008)
CV	−0.5*	73.9 (1901)	+0.9* (+0.2, +1.5)	91.2 (1918)	−5.5* (−9.1, −1.7)	55.5 (1924)	+0.8* (+0.7, +0.9)	85.3 (1977)	−3.8* (−4.0, −3.5)	26.5 (2008)
CBV	−1.8*	45.4 (1901)	+0.6* (+0.5, +0.8)	55.8 (1931)	−1.3* (−1.4, −1.1)	37.8 (1962)	−2.2* (−2.5, −1.8)	22.9 (1981)	−5.4* (−5.6, −5.2)	5.6 (2008)
TUM	+1.2*	27.7 (1901)	+0.8* (+0.6, +1.0)	36.5 (1929)	+2.1* (+2.0, +2.3)	82.2 (1965)	+1.0* (+0.7, +1.3)	101.9 (1988)	−2.6* (−2.9, −2.2)	61.3 (2008)
ACC	−0.6*	14.0 (1901)	+0.7* (+0.5, +0.9)	29.4 (1945)	−5.7 (−26.8, +21.5)	15.0 (1948)	+0.5 (−0.1, +1.0)	18.2 (1972)	−2.8* (−3.1, −2.5)	6.4 (2008)
END	−0.9#*	11.8 (1951)	−7.1* (−9.1, −5.1)	6.6 (1959)	−1.8* (−2.5, −1.2)	5.3 (1978)	+7.1 (−3.0, +18.3)	6.8 (1982)	−1.0*(−1.4, −0.6)	5.6 (2008)
NS	−0.8#*	5.6 (1951)	−3.7* (−4.3, −3.2)	2.7 (1968)	+0.6 (−0.1, +1.2)	3.4 (1985)	−0.5* (−0.9, −0.1)			2.8 (2008)
**B. Females**										
ALL	−1.7*	402.8 (1901)	−1.0* (−1.1, −0.8)	276.3 (1941)	−2.1* (−2.2, −2.0)					65.7 (2008)
RESP	−4.1*	89.4 (1901)	−1.6* (−2.1, −1.1)	49.6 (1938)	−9.0* (−11.3, −6.7)	12.3 (1952)	−3.7* (−3.9, −3.4)			1.8 (2008)
DIG	−1.6*	28.5 (1901)	−3.2* (−3.8, −2.5)	10.1 (1924)	+12.4 (−15.9, +50.1)	20.0 (1927)	−1.4* (−1.5, −1.2)	8.3 (1986)	−4.7* (−5.3, −4.8)	3.1 (2008)
INF	−4.4*	28.9 (1901)	−1.0* (−1.3, −0.8)	17.7 (1945)	−12.0* (−16.8, −6.9)	6.5 (1952)	−7.2* (−7.6, −6.9)	0.5 (1989)	+5.5* (+4.4, +6.5)	1.0 (2008)
CV	−2.0*	92.2 (1901)	−0.9* (−1.0, −0.8)	60.9 (1957)	−2.3* (−2.9, −1.8)	36.9 (1976)	−4.3* (−4.5, −4.1)			8.4 (2008)
CBV	−2.3*	32.5 (1901)	+0.6* (+0.5, +0.8)	46.6 (1932)	−1.1* (−1.5, −0.7)	39.5 (1950)	−3.0* (−3.2, −2.9)	14.6 (1980)	−5.6* (−5.8, −5.4)	3.2 (2008)
TUM	+0.1*	33.6 (1901)	+0.5* (+0.4, +0.6)	48.2 (1962)	−0.1 (−0.4, +0.2)	47.8 (1987)	−1.2* (−1.5, −0.8)			37.7 (2008)
ACC	−0.4*	4.6 (1901)	−0.1 (−0.4, +0.1)	3.9 (1950)	+0.7* (+0.2, +1.2)	5.3 (1980)	−3.4* (−3.9, −2.8)			1.9 (2008)
END	−1.9#*	9.7 (1951)	−4.5* (−6.3, −2.7)	7.1 (1960)	+5.0 (−14.5, +29.0)	8.2 (1963)	−1.4* (−1.8, −0.9)	5.7 (1987)	−3.2* (−3.7, −2.7)	3.0 (2008)
NS	−0.6#*	3.8 (1951)	−5.6* (−7.6, −3.5)	2.5 (1958)	−1.7* (−2.2, −1.2)	1.8 (1977)	+1.7* (+0.2, +3.3)	2.2 (1987)	−0.4 (−0.8, +0.1)	2.1 (2008)

*P<0.05; #Period 1951–2008.

**Table 3:** Age-adjusted mortality rates (per 100,000, world standard), for all causes and specific causes, and Annual Percent Changes (APC) between Join Points (JP) in Italian males (A) and females (B) aged 70 years and more during the period 1901–2008.

Causes of death	1901–2008 APC	Start (year)	APC1 (95% CI)	1^st^ JP (year)	APC2 (95% CI)	2^nd^ JP (year)	APC3 (95% CI)	3^rd^ JP (year)	APC4 (95% CI)	End (year)
**A. Males**										
ALL	−0.7*	524.3 (1901)	−0.2* (−0.3, −0.1)	530.4 (1944)	−5.2 (−16.3, +7.4)	427.2 (1947)	−0.3* (−0.5, −0.2)	378.2(1980)	−1.8* (−2.0, −1.6)	224.6 (2008)
RESP	−1.4*	92.8 (1901)	−0.4* (−0.7, −0.1)	79.3 (1939)	−4.4* (−5.7, −3.2)	34.4 (1954)	+0.5 (−0.3, +1.2)	41.8 (1976)	−2.2* (−2.6, −1.9)	20.0 (2008)
DIG	−0.8*	36.0 (1901)	−4.4* (−5.8, −3.0)	19.5 (1912)	−1.0* (−0.8, +1.2)	15.4 (1955)	+1.1* (+0.6, +1.6)	18.6 (1979)	−2.9* (−3.3, −2.6)	8.3 (2008)
INF	−1.9*	9.0 (1901)	−3.8* (−5.6, −2.0)	4.8 (1914)	+2.7* (+1.3, +4.1)	7.5 (1931)	−3.2* (−3.3, −3.0)	1.1 (1994)	+7.0*(+5.2,+8.8)	2.6 (2008)
CV	−0.1	88.3 (1901)	+1.7* (+1.0, +2.5)	111.8 (1917)	−2.9* (−5.3, −0.5)	85.3 (1925)	+1.2* (+1.1, +1.4)	151.5 (1973)	−2.5* (−2.8, −2.3)	63.2 (2008)
CBV	−0.8*	63.9 (1901)	+0.7* (+0.4, +1.0)	72.3 (1922)	+2.4* (+1.1, +3.7)	88.7 (1931)	−0.9* (−0.9, −0.8)	56.1 (1983)	−3.5* (−3.7, −3.3)	23.6 (2008)
TUM	+1.9*	12.9 (1901)	+2.5* (+1.7, +3.3)	18.2 (1914)	−0.6 (−1.7, +0.6)	14.6 (1925)	+2.7* (+2.6, +2.8)	70.9 (1981)	−0.2 (−0.4, +0.1)	67.5 (2008)
ACC	−0.4*	6.4 (1901)	+0.2 (0, +0.4)	6.6 (1950)	+2.2* (+1.6, +2.7)	11.4 (1974)	−1.5* (−1.9, −1.2)			6.5 (2008)
END	+0.6#*	9.3 (1951)	−6.6* (−8.9, −4.3)	6.4 (1958)	+0.2 (−0.3, +0.8)	6.3 (1978)	+8.8 (−0.8, +19.5)	9.1 (1982)	0 (−0.4, −0.3)	9.3 (2008)
NS	−1.5#*	5.0 (1951)	−6.1* (−7.8, −4.3)	3.0 (1960)	−0.8 (−1.6, +0.1)	2.6 (1976)	+6.5* (+4.2, +8.9)	4.8 (1985)	+2.2*(+1.7, +2.7)	7.6 (2008)
**B. Females**										
ALL	−1.2*	504.0 (1901)	+0.3 (−0.2, +0.8)	616.8 (1918)	−1.4* (−2.0, −0.9)	402.9 (1934)	+1.3 (−1.0, +3.6)	466.1 (1941)	−1.6* (−1.7, −1.6)	146.4 (2008)
RESP	−2.5*	91.9 (1901)	−0.6* (−0.9, −0.3)	70.6 (1941)	−8.6 (−22.7, +8.0)	47.3 (1945)	−3.1* (−3.3, −2.8)	9.5 (1995)	−0.9 (−2.6, +0.8)	8.4 (2008)
DIG	−1.2*	30.3 (1901)	−5.0* (−6.7, −3.3)	15.2 (1912)	−1.3* (−1.5, −1.1)	10.7 (1957)	−0.5* (−0.9, −0.1)	9.1 (1990)	−2.3* (−3.1, −1.5)	6.1 (2008)
INF	−2.4*	7.8 (1901)	0 (−0.3, +0.4)	5.5 (1944)	−11.2* (−16.8, −5.3)	2.4 (1951)	−4.0* (−4.3, −3.6)	0.5 (1991)	+9.4* (+7.9, +10.9)	2.0 (2008)
CV	−0.6*	103.0 (1901)	+1.2* (+0.7, +1.8)	132.1 (1918)	−3.3 (−6.7, +0.2)	93.5 (1924)	+0.8* (+0.6, +0.9)	132.0 (1967)	−2.7* (−2.8, −2.6)	45.3 (2008)
CBV	−0.8*	50.8 (1901)	+1.0* (+0.9, +1.2)	68.6 (1935)	−0.7* (−0.8, −0.5)	57.9 (1965)	−1.3* (−1.6, −1.0)	44.1 (1985)	−3.4* (−3.7, −3.2)	19.6 (2008)
TUM	+0.9*	13.5 (1901)	+0.4* (0, +0.7)	14.6 (1927)	+1.8* (+1.6, +2.1)	32.5 (1963)	0 (−0.1, +0.2)		-	32.6 (2008)
ACC	+0.7*	4.6 (1901)	−0.6* (−0.8, −0.3)	3.3 (1949)	−6.8* (+4.7, +9.0)	7.4 (1961)	+1.7* (−0.2, +3.1)	9.0 (1976)	−2.2* (−2.6, −1.8)	4.3 (2008)
END	+0.4#*	8.6 (1951)	−3.9* (−7.0, −0.7)	6.5 (1958)	+1.9* (+1.5, +2.4)	12.2 (1984)	−1.4* (−1.9, −0.9)			8.3 (2008)
NS	+1.7#*	3.5 (1951)	−4.2* (−5.1, −3.3)	2.0 (1965)	−0.4 (−1.8, 1.0)	1.9 (1977)	+7.2* (+4.3, +10.1)	3.5 (1985)	+3.0* (+2.5, +3.5)	6.4 (2008)

*P<0.05; #Period 1951–2008.

### Mortality by Causes, Gender and Age Groups

In 1901, INF, DIG, and RESP were by far the main causes of death in both genders of the 0–49 age class (Table 1). When considering the whole period, a statistically significant decrease was recorded for all diseases. There were two exceptions in males, including END, which did not vary significantly during 1951–2008, and TUM, which were slightly but significantly increased during the 1901–2008 period. S2–S5 Figures show the proportional mortality on all-age classes mortality for every single year of the 1901–2008 period, by considering 5 specific age sub-groups (0–4, 5–14, 15–29, 30–39, 40–49) within the 0–49 years group. In each graph, every bar represents the cumulative sum of the 10 causes of death. In the first two sub-groups (0–4 and 5–14), males and females have been merged due to the similarities in the distribution. The other sub-groups are represented separately by gender, due to the different behavior of distribution between genders.

In the 50–69-age class (Table 2), the inter-gender differences at the beginning of the 20th century were more evident. The highest mortality rates in males were attributable to RESP, followed by CV, CBV, DIG, INF, TUM, and ACC, whereas in females CV and RESP ranked first, followed by TUM, CBV, INF, DIG, and ACC. Mortality for TUM increased in both males and females during the 1901–2008 period, whereas all other diseases showed significant trends to a decline of mortality, which was particularly pronounced for RESP and INF.

In the ≥70 age class (Table 3), the prevailing causes of death in 1901 were RESP, CV, CBV, and DIG in males, whereas in females mortality for CV ranked first. Over the 1901–2008 period, mortality for CV was stable in old males and decreased in females. TUM and END significantly increased in both genders, and ACC decreased in males but increased in females. TUM were the only increasing cause of death for most of the period. The mortality for all other causes significantly decreased in both genders, but the decline for RESP, DIG, and INF was not as sharp as in the middle age and especially in the young age classes.

### JPLR Analysis of Variations of Specific Causes of Death over the 1901–2008 Period

**Figure 2:**
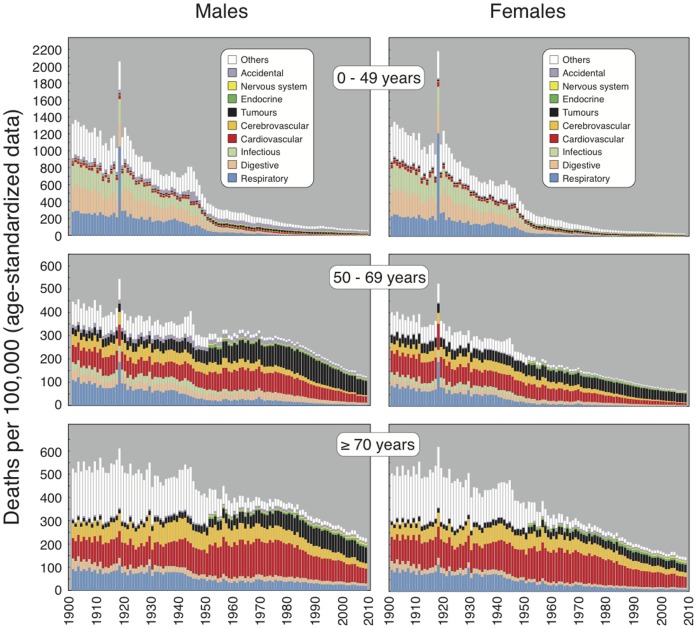
Age-standardized mortality data for the 10 main causes of death in Italy from 1901 to 2008 as related to gender and age classes.

As shown in Fig. 2 and detailed in Tables 1–3, contribution of OUC to all-cause mortality gradually decreased in both genders and all age classes, especially during the first half of the 20^th^ century. With few exceptions, the RESP were consistently decreasing with various paces. The decline of mortality for DIG was consistent throughout all JPs in the 0–49 year subjects of both genders, whereas in the 50–69 and ≥70 age classes there were periods either of decline or increase. Mortality for INF steadily decreased from 1901 to 1990 in males and 1992 in females and thereafter significantly increased until 2008 in all age classes.

A different picture emerged from the analysis of CDDs. In fact, mortality for CV was characterized by several fluctuations affecting the JPs detected by our analysis. Like for CV, the trends of mortality for CBV over the 1901–2008 period evolved through four JPs. Mortality trends for TUM were affected by both gender and age class. In males, a steady increase from 1901 to either 1977 (0–49 years) or 1988 (50–69 years) was followed by an evident decline until 2008. In old males there were two significant APCs, one negative (1901–1914) and one positive (1925–1983), whereas the other two APCs were not statistically significant. In 0–49 year females there was a positive APC lasting 15 years (1945–1970) but the other three periods had downward trends. In ≥50 year females there were three JPs only. In old women, a nonsignificantly positive APC (1901–1927) and a significantly positive APC (1927–1963) were followed by a long plateau period until 2008.

The situation was more complex for the diseases for which mortality data became available since 1950, especially for END. In the 0–49 years group of both genders, mortality data were quite unstable, with an alternation of upward and downward trends, whereas they were more stable in the 50–69 and ≥70 year groups. Mortality for NS showed in general a trend to decline in both genders, except significant and appreciable increases in the elderly after 1976–1977. Accordingly, during the last 30 years mortality for NS in the elderly increased 2.9 times in males and 3.4 times in females.

## Discussion

Striking mortality reductions have occurred during the 20th century in most countries in the world. The “epidemiological transition” theory suggests that cause-of-mortality patterns should shift from communicable diseases, which mainly affect infants and children, to CDDs at older ages [1], [25], [26]. This theory was conceived to explain the dynamic relationship between epidemiological trends and demographic changes. However, it has been argued that universalizing this theory only partially serves to explain mortality declines over the last century and eclipses key epidemiological differences related to socioeconomic status, race, and gender [27]. In fact, the health of a population is the product of the intersecting and dynamic influences from five different domains: genetic and gestational endowments, social circumstances, environmental conditions, behavioral choices, and medical care [28]. All the observed dramatic changes in the epidemiological scenario in Italy are the result of the interplay between composite genetic and environmental factors during the 20th century and the first years of the 21th century.

Interestingly, during the same period there was a significant association between the increase in caloric intake with time and the fall of mortality in the population, which in an ecological study reflects the increasing wellness in the Italian population, the progress in average nutritional status, lifestyle quality, socioeconomic level, and hygienic conditions [6]. For instance, as a proof of the amelioration of nutrition conditions, the height of Italians increased on an average 10 cm in one century [29]. Furthermore, as evaluated in European countries during the period 1900–2008, the prolongation of life expectancy is related to their economic growth [30].

Stratification according to three age classes of both all-cause mortality data and mortality due to the 10 main causes of death, along with the analysis of the time-related mortality fluctuations by means of JPLR, allowed us to draw an accurate and detailed picture of the epidemiological scenario evolution in Italy for more than one century as related to gender and age.

During the first half of the 20th century, the overall mortality decreased strongly in the youngest population, probably the most sensible to the changes in nutrition and wellness, while the welfare conditions of middle aged and elderly populations ameliorated more slowly. In the youngest group, the reduction of mortality has been continuing during the century, and this decrease has been steeper than those of the other age groups. This trend resulted in a crossing of the mortality curves of young individuals with those of the elderly in 1950 for males and in 1949 for females, and with those of the middle aged group in 1956 for males and 1965 for females. Thus, the most spectacular variations occurred in the 0–49 years population, with 21.3- and 39.6-fold falls in all-cause mortality during the period 1901-2008 in males and females, respectively. Within this age class, an important contribution was provided by infant mortality (0-1 years, see S1 Figure), which during the same period decreased almost 50 times, i.e. from 184.1‰ to 3.7‰ in males and from 149.4‰ to 3.2‰ in females [6].

Inter-gender variations involved all three age groups across the century. In the youngest, the differences were less pronounced compared to the older age groups. These patterns can be ascribed to the sharp decline in the mortality for INF, RESP, and DIG diseases due to the amelioration of nutritional conditions and dietary habits. In the second half of the century, formidable therapeutic and preventive measures, such as antibiotics and vaccines, were introduced. Moreover, these profound changes were combined with improved hygienic conditions and with changes in reproductive habits, involving a reduction in the number of children, which modified the fate of females relatively to pregnancy and delivery mortality.

Some social aspects, mainly affecting the mortality trends in the two older age groups, should also be taken into account. The differences between genders in occupational behavior and life habits were attenuated with time, particularly since the early Sixties. For most of the 20th century, occupational exposures and more risky lifestyles in men led to an advantage in mortality for women. In the last part of the 20th century, the homogenization of roles and lifestyles started to reduce the inter-gender differences, and the continuous improvement in the preventive, diagnostic and therapeutic strategies contrasted the mortality risks. In this context, the preventive actions addressed to fight hypertension, hyperglycemia, dyslipidemia, etc., together with new therapeutic tools, lowered the mortality risks in both genders, particularly for CV and CBV diseases, with a late effect also for TUM. The screening practices, mainly addressed to prevent female tumors, gave a further contribution in reducing mortality. Finally, the attention to the individual body care and health, usually more frequent in women, has become a common habit also for men in the last decades, which forecasts further changes in the mortality trends in the near future.

The causes of death entered into the statistics in 1951 showed conflicting trends by age. In fact, mortality for NS seemed to be mostly influenced by the increasing trends for neurodegenerative conditions such as Alzheimer, senile dementia and Parkinson's disease as related to ageing of population [31]. As to END fluctuations, the changes in ICD classification could be responsible for part of the trend variations. In fact, the sharp increase in mortality for END in the 0–49 years population, and especially in males from 1983–1984 to 1995, followed by a strong decrease until 1999, reflects the course of the AIDS epidemics in Italy (see S4 Figure). During the 1983–2008 period, the total number of AIDS deaths in Italy has been estimated to be 39,086 [32]. On the other hand, the slight decline of mortality for END in the ≥50 population may be related to the benefit of preventive measures towards the diabetic syndromes and other endocrine diseases, particularly of dietetic and pharmacological nature.

Finally, also the ACC, the only causes that are completely unrelated with the diet [6], were decreasing. The changes in ACC rates were very different by gender, so the differences over time were broader in males than in females and were probably due to increasing preventive measures introduced in life and occupational environments, particularly concerning measures aimed at preventing road accidents and their consequences [33]. The reduction was particularly evident in males belonging to the 0–49 age group.

## Conclusions

The results of this epidemiological study highlight the interplay between age and gender in affecting mortality data in the population. Inter-gender differences were negligible in younger people but became evident in the elderly, who are mainly affected by the balance between risk factors and protective factors involved in the determinism of CDDs.

## Dedication

MV, RL, RTM, SLM, and SDF wish to dedicate this paper to the memory of Alberto Quaglia, who passed away on May 6, 2014.

## Supporting Information

S1 Figure Breakdown of all-cause proportional mortality (%) within the 0–49 years age class during the 1901–2008 period.
(TIFF)

S2 Figure Proportional mortality by cause (%) during the 1901–2008 period in the mixed gender population from 0 to 4 years and from 5 to 14 years.
(TIF)

S3 Figure Gender-related proportional mortality by cause (%) during the1901–2008 period in the population aged 15 to 29 years.
(TIF)

S4 Figure Gender-related proportional mortality by cause (%) during the 1901–2008 period in the population aged 30 to 39 years.
(TIF)

S5 Figure Gender-related proportional mortality by cause (%) during the 1901–2008 period in the population aged 40 to 49 years.
(TIF)

S1 File Excel files reporting all the regression results as age-standardized death rates for males and females, separately for the three age classes.
(RAR)

S1 Appendix Information on references [7], [8], and [9].
(DOC)

S2 Appendix JPLR details.
(DOC)
