# Supplementary figures and images for: Age-Related Mortality Trends in Italy from 1901 to 2008
Source: PLoS One. 2014 Dec 8;9(12):e114027. doi: 10.1371/journal.pone.0114027 (PMC4259389; doi:10.1371/journal.pone.0114027)

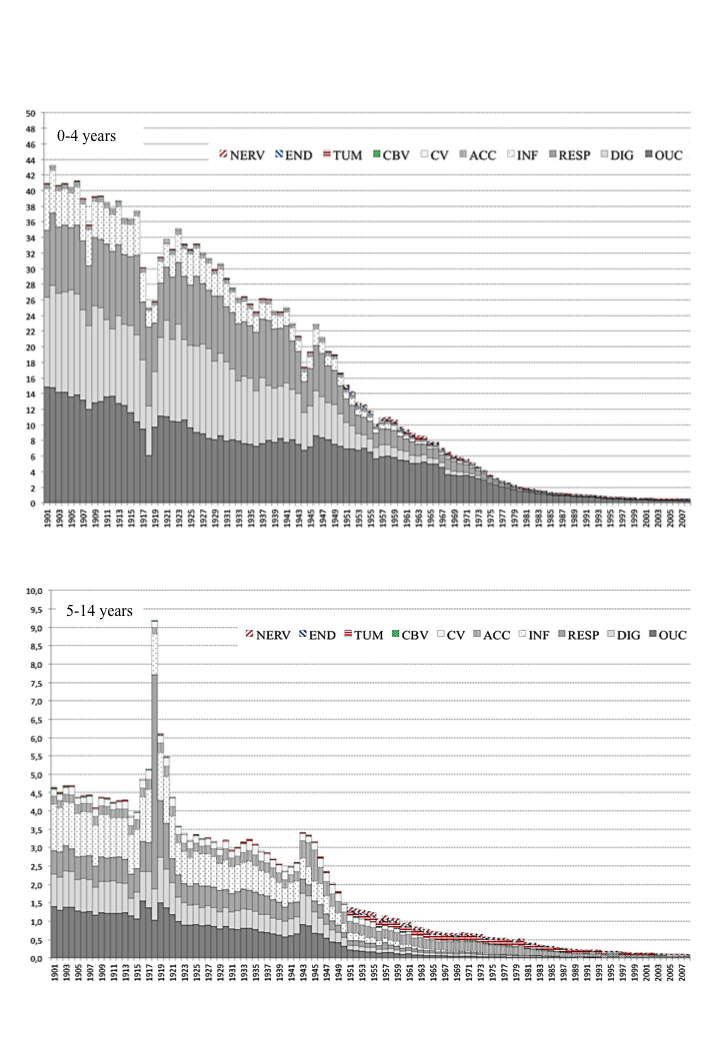

Supplement: S2 Figure — Proportional mortality by cause (%) during the 1901–2008 period in the mixed gender population from 0 to 4 years and from 5 to 14 years. (TIF) [file pone.0114027.s002.tif]

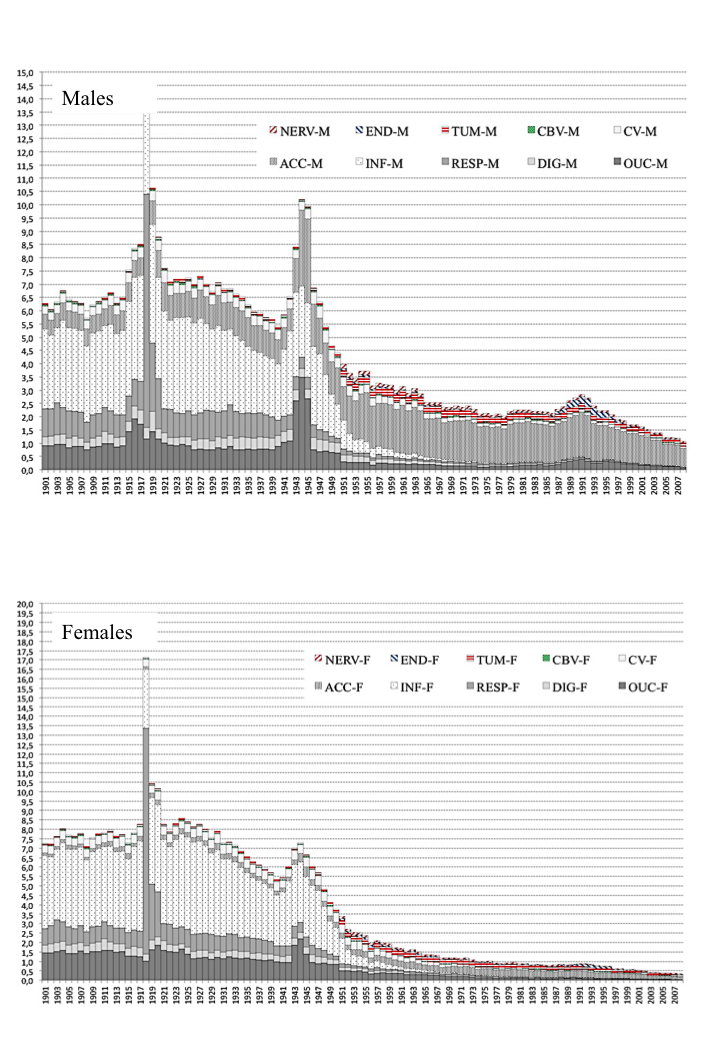

Supplement: S3 Figure — Gender-related proportional mortality by cause (%) during the1901–2008 period in the population aged 15 to 29 years. (TIF) [file pone.0114027.s003.tif]

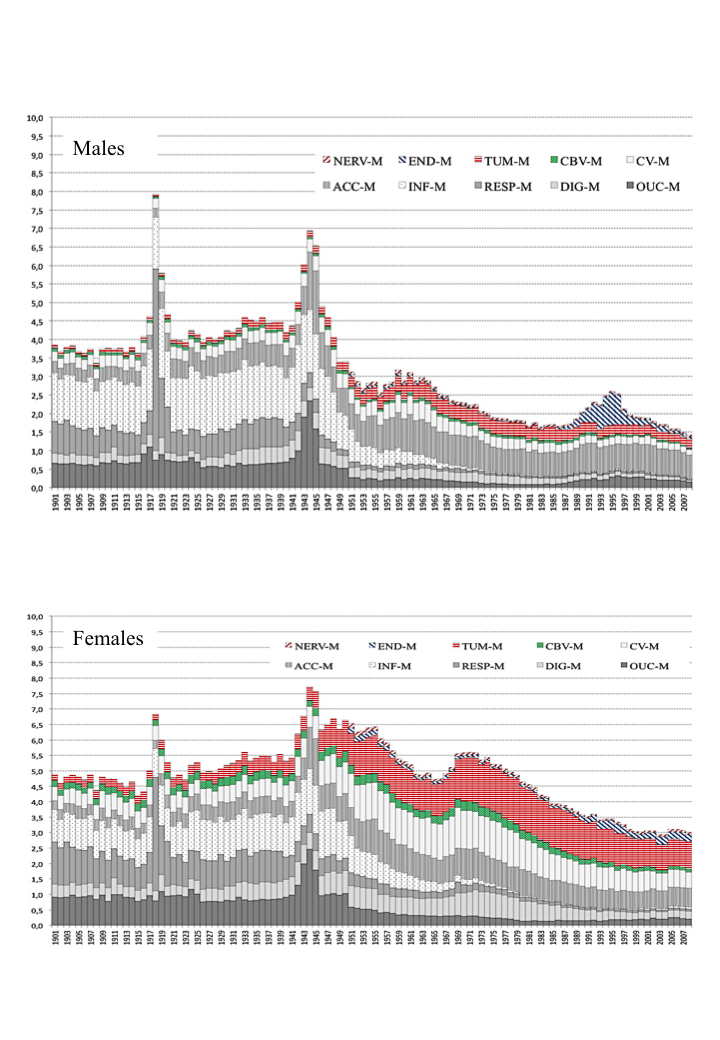

Supplement: S4 Figure — Gender-related proportional mortality by cause (%) during the 1901–2008 period in the population aged 30 to 39 years. (TIF) [file pone.0114027.s004.tif]

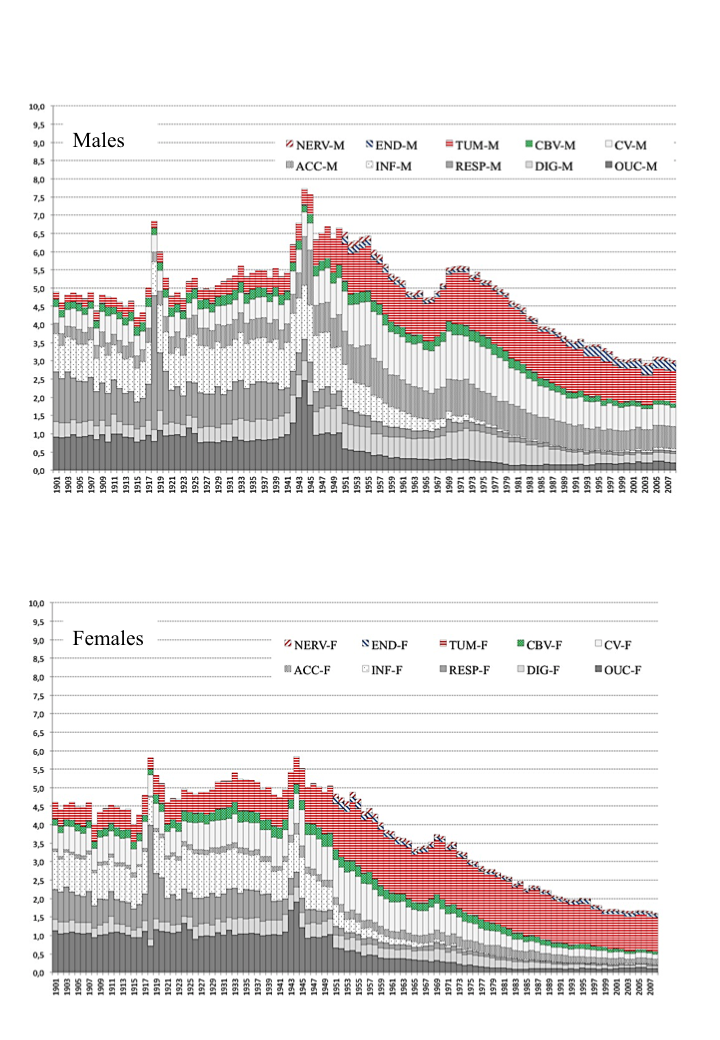

Supplement: S5 Figure — Gender-related proportional mortality by cause (%) during the 1901–2008 period in the population aged 40 to 49 years. (TIF) [file pone.0114027.s005.tif]
