# Supplementary material for: Age-Related Mortality Trends in Italy from 1901 to 2008
Source: PLoS One. 2014 Dec 8;9(12):e114027. doi: 10.1371/journal.pone.0114027 (PMC4259389; doi:10.1371/journal.pone.0114027)
Supplement: S1 Appendix — Information on references [7], [8], and [9]. (DOC) [file pone.0114027.s007.doc]

**Appendix S1**

Reference 7. The printed book “ISTAT Causes of Death 1887-1955” (1958) is no longer published, but can be found at the Library of the Statistics Office of Genova Municipality. The pages containing the mortality data used in the article range from page 12 to page 165. ([m.verdona@comune.genova.it](mailto:m.verdona@comune.genova.it)).

Reference 8. The printed book “ISTAT Historical Statistics of Italy 1861-1975” (1976) [“Istituto Centrale di Statistica, Sommario di Statistiche Storiche dell’Italia 1861-1975”] is no longer published, but can be found at the Library of the ISTAT National Archive in Rome. The table pertaining the article is 27. The book can be downloaded at the following link:

[http://lipari.istat.it/digibib/Sommario%20statistiche%20storiche/TO00159260Sommario%20di%20statistiche%20storiche%20dell'Italia%201861-1975+ocr%20ottimizzato.pdf](http://lipari.istat.it/digibib/Sommario statistiche storiche/TO00159260Sommario di statistiche storiche dell'Italia 1861-1975+ocr ottimizzato.pdf)

Reference 9. The tables in “ISTAT Health for All” database are defined by the main code “02 Mortality by causes” and range from sub-code 1000 to sub code 1960.
