# Supplementary material for: Age-Related Mortality Trends in Italy from 1901 to 2008
Source: PLoS One. 2014 Dec 8;9(12):e114027. doi: 10.1371/journal.pone.0114027 (PMC4259389; doi:10.1371/journal.pone.0114027)
Supplement: S2 Appendix — JPLR details. (DOC) [file pone.0114027.s008.doc]

**Appendix S2**

The JoinPoint Linear Regression (JPLR) model has the following characteristics. Log-linear model:

ln(y) = βx + e

in the hypothesis of a constant variance. This selection assumes that the random errors in the regression model are homoscedastic (i.e., have a constant variance) and estimates the regression coefficients by ordinary least squares.

The algorithm produces a number of models corresponding to the maximum number of joinpoints. For choosing the best model we used the sequence of permutation tests, which ensures that the approximate probability of overall Type I error is less than the specified significance level (also called the alpha level, default = 0.05). Assuming that the default value of the minimum number of joinpoints is 0, "the overall Type I error" is the probability of incorrectly concluding that the underlying model has one or more joinpoints when, actually, the true underlying model has no joinpoints. The program performs permutation tests to select the number of joinpoints. Since fitting all N! possible permutations of the data would take too long, the program takes a Monte Carlo sample of these N! data sets, using a random number generator. The significance level is set at p < 0.05.
